# Plasma ctDNA RAS mutation analysis for the diagnosis and treatment monitoring of metastatic colorectal cancer patients

**DOI:** 10.1093/annonc/mdx125

**Published:** 2017-04-13

**Authors:** J Vidal, L Muinelo, A Dalmases, F Jones, D Edelstein, M Iglesias, M Orrillo, A Abalo, C Rodríguez, E Brozos, Y Vidal, S Candamio, F Vázquez, J Ruiz, M Guix, L Visa, V Sikri, J Albanell, B Bellosillo, R López, C Montagut

**Affiliations:** 1 Cancer Research Program, FIMIM Hospital del Mar, Barcelona, Spain;; 2 Medical Oncology Department, Hospital del Mar, Barcelona;; 3 Traslational Medical Oncology Group (Oncomet)/Liquid Biopsy Analysis Unit, Health Research Institute of Santiago (IDIS), Complexo Hospitalario Universitario de Santiago de Compostela (SERGAS) CIBERONC, Santiago de Compostela;; 4 Pathology Department, Hospital del Mar, Barcelona;; 5 Sysmex Inostics Inc., Mundelein, USA;; 6 Universitat Pompeu Fabra, Barcelona, Spain

**Keywords:** ctDNA, RAS mutations, colorectal cancer, liquid biopsy, tumor dynamics, heterogeneity

## Abstract

**Background:**

*RAS* assessment is mandatory for therapy decision in metastatic colorectal cancer (mCRC) patients. This determination is based on tumor tissue, however, genotyping of circulating tumor (ct)DNA offers clear advantages as a minimally invasive method that represents tumor heterogeneity. Our study aims to evaluate the use of ctDNA as an alternative for determining baseline *RAS* status and subsequent monitoring of *RAS* mutations during therapy as a component of routine clinical practice.

**Patients and methods:**

*RAS* mutational status in plasma was evaluated in mCRC patients by OncoBEAM™ RAS CRC assay. Concordance of results in plasma and tissue was retrospectively evaluated. *RAS* mutations were also prospectively monitored in longitudinal plasma samples from selected patients.

**Results:**

Analysis of *RAS* in tissue and plasma samples from 115 mCRC patients showed a 93% overall agreement. Plasma/tissue *RAS* discrepancies were mainly explained by spatial and temporal tumor heterogeneity. Analysis of clinico-pathological features showed that the site of metastasis (i.e. peritoneal, lung), the histology of the tumor (i.e. mucinous) and administration of treatment previous to blood collection negatively impacted the detection of *RAS* in ctDNA. In patients with baseline mutant *RAS* tumors treated with chemotherapy/antiangiogenic, longitudinal analysis of *RAS* ctDNA mirrored response to treatment, being an early predictor of response. In patients *RAS* wt, longitudinal monitoring of *RAS* ctDNA revealed that OncoBEAM was useful to detect emergence of *RAS* mutations during anti-EGFR treatment.

**Conclusion:**

The high overall agreement in *RAS* mutational assessment between plasma and tissue supports blood-based testing with OncoBEAM™ as a viable alternative for genotyping *RAS* of mCRC patients in routine clinical practice. Our study describes practical clinico-pathological specifications to optimize *RAS* ctDNA determination. Moreover, OncoBEAM™ is useful to monitor *RAS* in patients undergoing systemic therapy to detect resistance and evaluate the efficacy of particular treatments.

## Introduction

Monoclonal antibodies (moAb) directed against EGFR—cetuximab and panitumumab—are standard components of treatment regimens for metastatic colorectal cancer (mCRC) patients, either alone or in combination with chemotherapy. The current standard of care is to determine mutations in *RAS* in all mCRC tumors before initiating treatment, as critical biomarkers of innate resistance to anti-EGFR [1]. Moreover, all mCRC patients that initially respond to anti-EGFR therapy eventually develop resistance, which in ∼50% of cases is due to the emergence of *RAS* mutations [2–5]. Currently, *RAS* mutation determination is carried out in formalin fixed paraffin-embedded samples from tumor tissue. 

Circulating DNA fragments carrying tumor specific sequence alterations (circulating tumor DNA, ctDNA) are found in the cell-free fraction of blood, representing a variable and generally small fraction of the total circulating cell-free DNA (cfDNA). Tumor genotyping using ctDNA offers potential advantages particularly in the metastatic setting as a safe minimally invasive alternative to tissue [3].

Prior studies have demonstrated a high degree of concordance between somatic mutations detected in tumor tissue and those determined in ctDNA of patients with advanced tumors [6, 7]. The use of ctDNA has also demonstrated utility to predict treatment response to chemotherapy. Previous ctDNA studies utilized massively parallel (direct) sequencing of tumor tissue in order to identify somatic alterations specific to individual patients, which were subsequently incorporated into the development of a personalized gene panel to detect these mutations in blood samples. Although useful in a research setting, a personalized NGS panel approach is currently not amenable to routine clinical practice in that it requires significant dedicated resources in highly qualified research laboratories. Alternatively, blood-based tests that encompass a panel of the most frequently occurring mutations for a given tumor type and which can be used to interrogate the plasma of patients with high sensitivity present a practical approach for routine clinical care. The first and only test thus far for the determination of RAS mutations in ctDNA with European Conformity (CE-marked) *in vitro* diagnostic (CE-IVD) is the OncoBEAM RAS CRC assay, which detects 34 mutations in exons 2, 3, and 4 in the *KRAS* and *NRAS* genes as recommended by current clinical practice treatment guidelines (NCCN, ESMO, EMA).

The aim of the present study was to evaluate the clinical applications of the OncoBEAM RAS CRC assay in routine clinical practice for the diagnosis, assessment of response to chemotherapy/antiangiogenic treatment and monitoring of acquired resistance to anti-EGFR therapy in mCRC patients.

## Materials and methods

### Study design and sample collection

A retrospective-prospective study was carried out in two Spanish Institutions. Patients with histologically confirmed metastatic colorectal cancer and anti-EGFR treatment naïve were eligible for the study. Blood samples were collected in all patients before the administration of anti-EGFR treatment. For those patients undergoing monitoring, serial blood samples were collected every 4 weeks coinciding with the treatment visit and at the moment of progressive disease. See full inclusion criteria and regulatory aspects in supplementary material, available at *Annals of Oncology* online.

OncoBEAM™ RAS CRC assay was used to detect *RAS* mutations in plasma, and *RAS* mutation detection in tissue samples were carried out according to standard-of-care (SoC) procedures validated by each hospital (details in supplementary material and Table S4, available at *Annals of Oncology* online).

### Statistical analysis

Variables were described using median and interquartile range (IQR) when continuous, and percentage when categorical. For mutant allele fraction (MAF) levels comparisons between different groups regarding clinical variables, we carried out Mann–Whitney *U* test for dichotomic variables and Kruskal–Wallis test for polycothomic variables. Tests were carried out under SPSS v.22 with a significance level of *P* < 0.05. Graphics were built using R 3.3.1.

## Results

### Patient characteristics and concordance of extended RAS determination in plasma versus tissue

**Figure 1.**
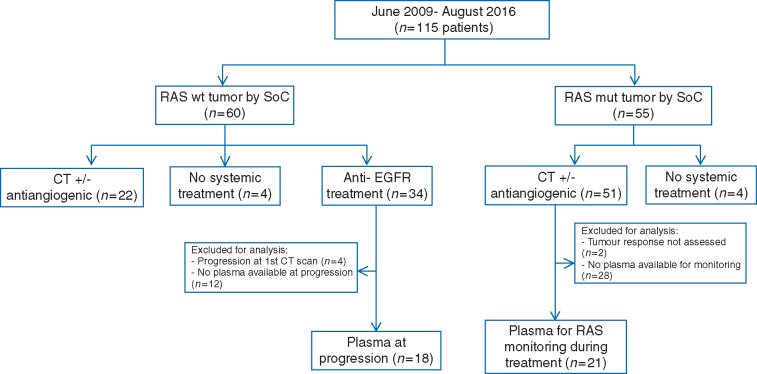
Study flowchart. Number of patients included in each of the analysis endpoints and reasons for exclusion are depicted.

From June 2009 to August 2016, 115 patients with mCRC were included, all of them had at least one baseline blood draw. Study flowchart is presented in Figure 1. Clinico-pathological characteristics of the patients are described in supplementary Table S1, available at *Annals of Oncology* online. At the time of basal ctDNA collection, all patients were naïve to anti-EGFR treatment and 82 patients (71%) had not received any therapy in the metastatic setting. The median time from tumor tissue specimen collection to ctDNA collection was 47.5 days (range 0–1783 days) in therapy-naïve patients.

**Figure 2.**
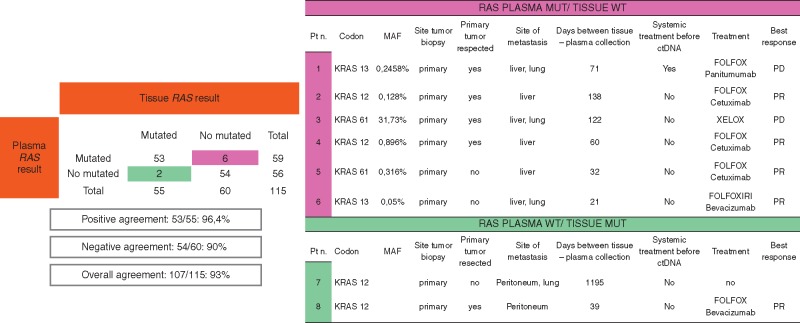
Comparison of RAS mutations detected in tissue versus plasma and analysis of discrepancies. Overall concordance analysis between *RAS* mutations detected in tumor by SoC and BEAMing plasma. Positive agreement (patients RAS mutated in plasma and tissue analysis) and negative agreement (patients wild-type in tissue and plasma). Clinico-pathological and treatment characteristics from eight discordant cases.

Of the 115 patients included in the study, 55 (47.8%) and 59 (51.3%) were shown to have *RAS* mutations in their tumor samples as detected by SoC *RAS* tissue testing and as detected in ctDNA by RAS OncoBEAM, respectively (supplementary Figure S1, available at *Annals of Oncology* online). The overall concordance of *RAS* results between ctDNA RAS OncoBEAM assay and standard techniques for tissue analysis was 93% (107/115 patients), kappa index 0.844 (95% CI 0.746–0.941) (Figure 2).

### Characterization of RAS tissue versus RAS plasma discordant cases

Among 55 patients in whom a *RAS* mutation was detected in tissue, 53 also had a *RAS* mutation in plasma (positive percentage agreement, PPA of 96.4%) (Figure 2). There were only two cases with *RAS* mutated tissue in whom no *RAS* mutation was detected in ctDNA. Both had localized tumors at the time of diagnosis that were initially removed. At relapse, when ctDNA extraction was carried out, both patients had minimal tumor burden: one with only peritoneum metastasis and the other had one infra-centimetric lung metastasis and one single implant in the peritoneum.

Among 60 patients determined to be *RAS* wt in tissue, no *RAS* mutations were observed in ctDNA in 54 cases (negative percentage agreement, NPA of 90%). In the remaining 6 patients, plasma RAS BEAMing detected a *RAS* mutation that SoC tissue testing had not revealed. In all of these cases, the primary tumor served as the source for *RAS* mutational analysis and had been removed before ctDNA sampling. Notably, all six patients had, at least, liver metastasis when blood was drawn for ctDNA analysis.

**Figure 3.**
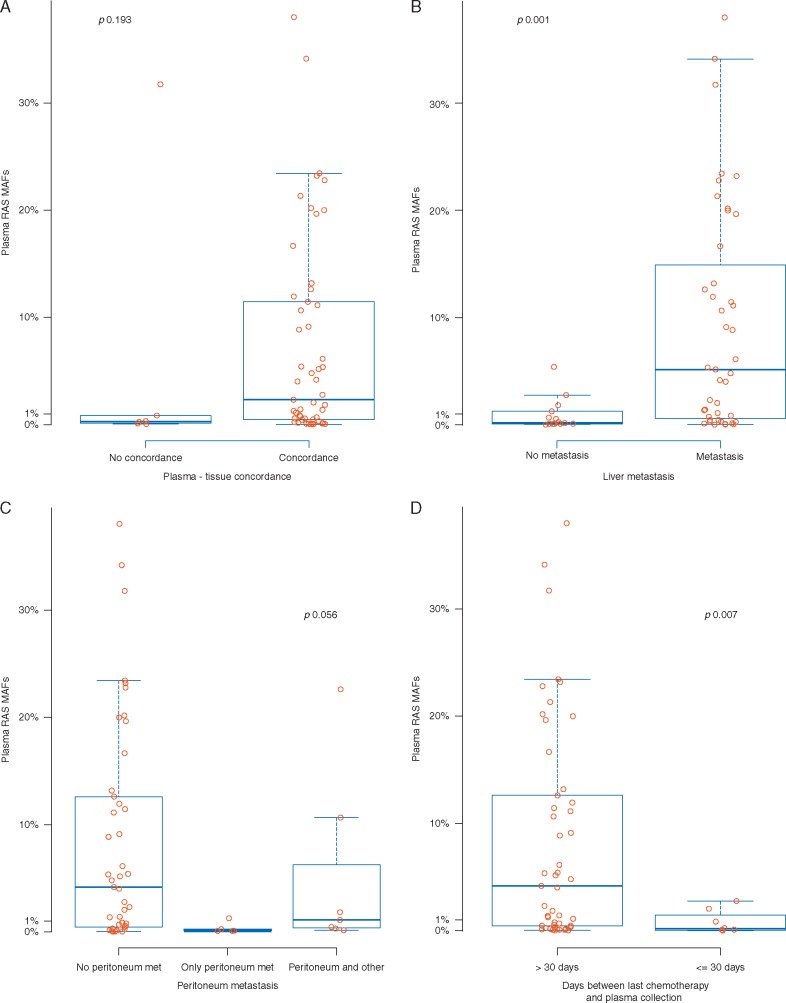
Correlation of circulating RAS mutations and clinico-pathological characteristics. (A) Differences in *RAS* ctDNA mutant allele fraction (MAF) in patients with concordant *RAS* plasma and tissue determination compared with discordant cases (*RAS* wild-type in plasma and *RAS* mutated in tissue or *RAS* mutated in plasma and *RAS* wild-type in tissue). (B) Differences in *RAS* ctDNA MAF according to patients with liver metastasis versus patients without liver involvement. (C) Differences in *RAS* ctDNA MAF according to patients without peritoneum involvement, patients with only peritoneum metastasis and patients with peritoneum plus another metastatic site. (D) Differences in *RAS* ctDNA MAF according to treatment naïve patients versus patients with previous systemic treatment received within a month prior ctDNA blood extraction.

Interestingly, in five out of the six *RAS* tissue-/plasma+ discordant cases, the *RAS* MAF detected in ctDNA was under 1%. We compared the MAF of concordant and discordant cases. As shown in Figure 3A, there was a trend towards lower *RAS* plasma MAF in discordant cases compared with patients with concordant *RAS* in tissue and plasma (median *RAS* MAF 0.281% and 2.317%, respectively; *P*=0.193).

### Differences in RAS ctDNA MAF according to clinico-pathological characteristics

The global median *RAS* plasma MAF was 1.84% (IQR: 0.284–11.290) (supplementary Table S1, available at *Annals of Oncology* online). No differences in MAFs were observed in relation to age, gender, initial stage at diagnosis or primary site of disease (right versus left).

While no differences in *RAS* MAF were seen in relation to the number of metastatic sites, differences were observed depending on the site of metastasis location (Figure 3B and C). Patients with liver involvement had higher *RAS* ctDNA compared with those without liver metastases (4.806% versus 0.203%; *P*=0.001). In contrast, MAF from patients having only peritoneum metastases was lower (0.1%) than patients without peritoneum involvement (4.026%), than patients with peritoneum metastasis in addition to at least one other metastatic site (1.109%; *P *=0.056). MAF was lower in patients with only lung metastatic involvement (0.033%). Of note, in two patients that presented with tumors having mucinous histology MAF was below the median (0.451% and 0.161%, respectively; *P *<* *0.05).

We then sought to analyze the impact of treatment on *RAS* mutation detection in plasma. No differences were observed in *RAS* plasma MAF between patients in whom the primary tumor had been removed before basal ctDNA extraction (4.026%) and patients in whom ctDNA was extracted from blood drawn at the time primary tumors had not been resected (1.558%; *P*=0.584). Regarding the relation between systemic treatment and plasma *RAS* mutation detection, 8 of 59 *RAS* mutant patients (13.6%) had received previous treatment with chemotherapy (comprising 5FU, oxaliplatin and/or irinotecan) ± anti-VEGF within a month prior ctDNA blood extraction. In all of these patients, lower *RAS* plasma MAFs were observed (0.173%; range 0.074–1.156) when compared with treatment-naïve patients (4.178%; range 0.451–12.620; *P*=0.007) (Figure 3D), emphasizing the relevance of blood draw timing for an accurate RAS determination.

### Response to anti-EGFR therapy and prognosis according to baseline RAS ctDNA determination

We then determined the impact of *RAS* detection in predicting response to anti-EGFR based therapy. Among 54 patients having *RAS* wt tumor tissue, 34 were treated with anti-EGFR monoclonal antibodies (31 with cetuximab and 3 with panitumumab-based regimens; 30 plus chemotherapy and 4 in monotherapy). Twenty-three achieved a complete or partial response (68%) and 7 patients (20%) had stable disease for more than 16 weeks. Among tissue *RAS* wt patients treated with anti-EGFR therapy, four were found to have a plasma *RAS* positive result (Figure 2, discordant patients #1, #2, #4, #5). Three of them achieved a partial response, whereas one showed progressive disease after administration of anti-EGFR treatment.

Despite the retrospective nature of our study, we determined the progression-free survival (PFS) according to *RAS* mutation determination in plasma and *RAS* mutation determination in tissue. PFS was 10.3 month (95% CI 7.7–25) for wt *RAS* tissue patients and 10.3 months (95% CI 7.7–19.8) for RAS wt plasma patients.

In addition, baseline high *RAS* ctDNA MAF have been associated with low survival [8]. We analyzed the prognosis impact of basal MAF levels in a cohort of 22 patients with at least 3 year of follow-up. Patients with MAF levels ≥1% had significant lower PFS and OS than those with basal levels <1% (supplementary Figure S2, available at *Annals of Oncology* online). These data suggest that ctDNA levels could also provide valuable information to predict the disease evolution in *RAS* mutant patients before treatment onset.

### Longitudinal ctDNA RAS testing for assessing response to patients treated with systemic treatment

Because ctDNA analysis has the capacity to reflect tumor load [2], we examined the utility of OncoBEAM RAS CRC ctDNA testing to monitor the efficacy of response of patients to treatment.

RAS was longitudinally monitored in serial blood draws from 21 patients with baseline RAS mutations undergoing systemic therapy. Seven patients were treated with combination chemotherapy + antiangiogenic therapy, 12 received chemotherapy alone and 2 anti-EGFR-based treatment (supplementary Table S2, available at *Annals of Oncology* online). A first CT-scan was carried out concurrent with the ctDNA RAS monitoring to evaluate tumor response after 8–12 weeks of treatment. Analysis of RAS ctDNA at the time of this first CT-scan revealed a dramatic decrease in plasma RAS MAF in responding patients with a median of 100%. For patients with clinical response to treatment, no differences in MAFs were observed in relation to the type of response achieved (median MAF reduction in patients with SD 99% versus 100% in patients with PR; *P* = 0.21). However, MAF percentage of change was significantly lower in patients that progressed at first evaluation of response compared with patients with clinical benefit (PR + SD) (132% increase versus 99% reduction, respectively, *P* = 0.027)

**Figure 4:**
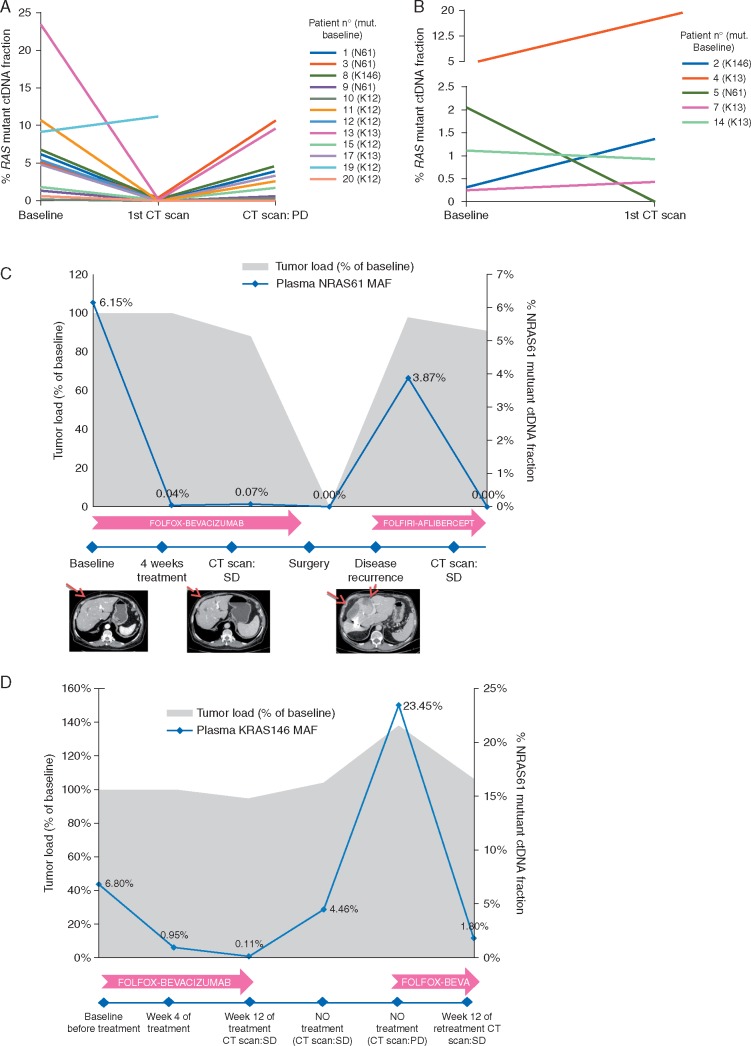
Longitudinal analysis of plasma RAS ctDNA to evaluate response to treatment with chemotherapy** ± **antiangiogenic. (A) *RAS* ctDNA dynamics in nine patients with *RAS* mutated tumors treated with chemotherapy** ± **antiangiogenic that initially respond to treatment. Frequency of circulating *RAS* mutant alleles at baseline, at time of first CT-scan to evaluate treatment response and at disease progression. Decline and increase in circulating *RAS* MAF correlate with response and progression to treatment, respectively. (B) *RAS* ctDNA dynamics in 5 patients with *RAS* mutated tumors that progressed at first CTscan at 8–12 weeks from beginning of treatment. (C) Patient diagnosed with stage IV rectal cancer with liver metastasis. An *NRAS* codon 61 mutation was detected in tissue and plasma. After 4 weeks of treatment with FOLFOX + bevacizumab, plasma MAF dramatically decreased correlating with a stable disease observed in the CT-scan at week 12. The patient underwent surgery of the primary tumor and liver metastasis, and plasma *RAS* became undetectable. Eight months later, the patient relapsed and *RAS* MAF increased accordingly. Three months after initiating second line treatment with FOLFIRI + aflibercept, the patient achieved a stable disease by CT scan, and no plasma ctDNA *RAS* mutations were detected. (D) Monitoring ctDNA KRAS codon 146 mutation during treatment with FOLFOX-Bevacizumab in patient #3, diagnosed with a stage IV colon cancer with lung and liver metastasis. The colonoscopic biopsy analysis was *RAS* wt but plasma ctDNA showed a *KRAS* codon 146 mutation. Following removal of the primary tumor, re-analysis of *RAS* in the surgical sample confirmed the plasma result. The patient received FOLFOX + bevacizumab with an early decrease in *RAS* ctDNA that became undetectable at 12 weeks, alongside at the first CT scan. Treatment was discontinued and a subsequently increase in KRAS codon 146 MAF was observed, which then rapidly decreased when the chemotherapy was reintroduced. Gray area indicates tumor load. Blue line indicates changes in ctDNA KRAS146 frequency.

In 10 out of 11 responding patients that subsequently progressed, *RAS* ctDNA MAF increased accordingly, though in most cases patients exhibited lower MAFs than at the time of diagnosis (Figure 4A; supplementary Table S2, available at *Annals of Oncology* online). Of note, in one patient having a basal *KRAS* mutation in codon 12 (basal RAS MAF 9.12%), a decrease in *RAS* MAF was initially observed that was quickly followed by an increase in *RAS* MAF at week 12 although the CT scan showed stable disease. This patient subsequently showed a rapid progression of disease and died 4 months later.

Representative time courses of ctDNA along with clinical and radiologic data on two subjects are provided (Figure 4C and D), showing the high accuracy of *RAS* plasma ctDNA dynamics as a surrogate marker of tumor load and a potential tool to evaluate early response to treatment.

### ctDNA extended RAS for monitoring RAS mutations during and after withdrawal of anti-EGFR therapy

We and others previously reported that acquired resistance to anti-EGFR treatment is linked to the emergence of *RAS* mutations, that can be tracked in the blood of patients [2, 3, 8, 9]. In our study, we examined the value of OncoBEAM RAS CRC testing to detect the emergence of *RAS* mutations during anti-EGFR treatment. Plasma was available at the time of disease progression from 18 cases with acquired resistance to anti-EGFR therapy (i.e. disease progression after an initial complete response, partial response or stable disease for more than 16 weeks). Emergence of *RAS* mutations was detected in 7/18 patients (39%), 5 of them treated with chemotherapy + anti-EGFR and two with anti-EGFR monotherapy (supplementary Table S3, available at *Annals of Oncology* online). The most frequent mutations involved *KRAS* codon 12, *KRAS* codon 13 and *NRAS* codon 61. In three cases, different *RAS* mutations were concomitantly detected. Median *RAS* MAF detected at the time of anti-EGFR progression was 2.17% (range 0.024–24.957).

## Discussion

Our study demonstrates that OncoBEAM RAS CRC assay is an efficient and accurate tool to be used in routine clinical practice with several applications in mCRC patients, including determination of baseline *RAS* at diagnosis to decide anti-EGFR therapy, assessment of efficacy to treatment and monitoring of the emergence of *RAS* mutations as a mechanism of resistance to anti-EGFR therapy.

The high overall agreement between baseline plasma and tissue RAS mutation status demonstrated in more than 100 patients evaluated in our study supports the use of blood-based testing with OncoBEAM™ RAS CRC as a viable alternative to tissue SoC for determining *RAS* mutation status in mCRC patients treated in routine clinical practice. Previous studies have shown that ctDNA can be detected in patients with mCRC by using personalized research panels with dPCR [7, 10, 11]. Recent publications have also shown a very high sensitivity with BEAMing to detect ctDNA mutations [12, 13]. However, to the best of our knowledge, this is the first study that explores the clinical use of plasma *RAS* determination by using a CE-marked assay in a daily clinical routine setting and in a large real world cohort of patients. Moreover, the minimal level of discordance (6%) between *RAS* tissue and plasma detection shown in our study is acceptable from a clinical point of view. In fact, it is far lower than the 5%–20% discrepancy found in *RAS* mutation detection when comparing two different tissue RAS testing SoC techniques [14, 15].

In an effort to explain plasma/tissue discrepancies as well as to better understand the biology of circulating tumor DNA, we identified several clinico-pathological features linked to low *RAS* ctDNA detection, including peritoneal/lung metastases or mucinous histology. In contrast, no correlation was found between the number of metastasis and *RAS* ctDNA mutations in our study. This data suggests that intrinsic biological characteristics of the tumor rather than tumor burden may impact ctDNA release. In order to appreciate the utility and further optimize the routine evaluation of *RAS* ctDNA determination in daily clinical practice, we also studied external factors that may influence the result of *RAS* ctDNA determination. We found that the administration of recent systemic treatment had a clear negative impact on the ability to detect *RAS* mutations in the blood of patients, emphasizing the importance of collecting plasma for basal *RAS* analysis before the initiation of any systemic treatment. On the contrary removal of the primary tumor before blood draw for *RAS* analysis did not impact the *RAS* mutation results. In global, our study shows that the pattern of genetic alterations in cancer patients is dynamic and is affected by intrinsic and extrinsic factors.

Importantly, we also found a potential role of OncoBEAM RAS ctDNA assay in monitoring response and resistance during treatment. In patients with *RAS* mutant tumors, *RAS* plasma mirrored clinical and radiological response to treatment with chemotherapy drugs and was an early predictor of response. Likewise, Tie et al. [10] reported changes in ctDNA for mCRC patients treated with chemotherapy, although a more complex research-based approach was used. Moreover, we showed a potential use of *RAS* ctDNA in evaluating response to antiangiogenic drugs, which could be complementary to RECIST. On the other hand, in patients with *RAS* wt tumors treated with anti-EGFR, OncoBEAM RAS CRC was a valid tool to detect *RAS* mutations of resistance.

Despite the great value of the results presented, there are several limitations to our study. It is a retrospective analysis. Longitudinal blood extractions were only carried out in a limited number of patients. Additionally, given the low number of patients with specific clinico-pathological characteristics, our inferences from associations with *P*-values marginally <0.05% should be cautiously interpreted.

Overall, our data show that the OncoBEAM RAS CRC assay offers a minimally invasive and highly sensitive method for *RAS* assessment in plasma of mCRC patients which can be readily implemented into routine clinical practice to perform baseline diagnosis to select candidate patients to anti-EGR therapy. Moreover, we show a potential use of OncoBEAM RAS in assessing the dynamics of *RAS* to monitor response and resistance to treatment practice.

## Supplementary Material

mdx125_supp
