# Supplementary material for: Plasma ctDNA RAS mutation analysis for the diagnosis and treatment monitoring of metastatic colorectal cancer patients
Source: Ann Oncol. 2017 Apr 13;28(6):1325–32. doi: 10.1093/annonc/mdx125 (PMC5834035; doi:10.1093/annonc/mdx125)
Supplement: mdx125_supp [file mdx125_supp.zip › Supplementary M&M.docx]

**Study design and population**

The study was approved by the local institutional research ethics committee (CEIC-012/4741/I and CEIC-2009-289) and all participating patients provided written informed consent. Part of tissue samples from patients included in this study were provided by the Biobank of I.D.I.S.-C.H.U.S. (PT13/0010/0068), integrated in the Spanish National Biobank Network and they were processed following standard operating procedures with the appropriate approval of the Ethical and Scientific Committees.

Tumor burden, metastasis location and therapy response were evaluated with standard imaging procedures (computed tomography, CT) by a specialized radiologist. CT of the chest, abdomen and pelvis was performed every three months and evaluated for disease response according to the Response Evaluation Criteria in Solid Tumors (RECIST), version 1.1.

**Sample collection and *RAS* mutational status analysis**

Blood samples for *RAS* testing using the OncoBEAM RAS CRC assay were collected in K2 EDTA tubes (10ml), processed into plasma within 2h (1,800 x g  for 10 minutes at 18–23°C), and stored at −80 °C until ctDNA analysis. For most patients, 2 mL and in one case 0,5 mL of plasma were used for DNA purification. DNA was extracted using the QIAamp DNA Circulating Nucleic Acid Kit (Qiagen, Venlo, Netherlands) according to the manufacturer's instructions. ctDNA isolation from plasma and BEAMing assays were conducted by Hospital del Mar (Barcelona), Complexo Hospitalario Universitario de Santiago de Compostela (Liquid Biopsy analysis Unit) and Sysmex Inostics GmbH (Hamburg, Germany), as previously reported (1). Using the OncoBEAM™ RAS CRC assay we analysed a total of 34 RAS mutations, including 16 *KRAS* mutations and 18 *NRAS* mutations within exons 2, 3 and 4 (Supplementary Table S4). The cutoff thresholds for the BEAMing assay of plasma DNA were standardized for each codon.

The percentage of mutant allelic fractions (MAFs) were calculated as the fractional abundance of mutant DNA alleles relative to wild-type DNA alleles in a plasma sample. The absolute number of *RAS* mutant alleles are not reported by BEAMing as the determination of mutant status is dependent on the total amount DNA in an individual sample. Total circulating DNA levels (both wild-type and mutant) are subject to interpatient variability which may be directly related to tumor burden or other characteristics such as inflammation and immune response.

*RAS* mutation detection in tissue samples for all patients were performed according to standard-of-care (SoC) procedures validated by each hospital; RAS tissue testing was performed using the Therascreen KRAS RGQ PCR kit (Qiagen, Hilden, Germany), COBAS KRAS mutation test (Roche Diagnostics, Rotkreuz, Switzerland) and pyrosequencing (PyroMark Q24, Qiagen). For discrepant cases wherein plasma and tissue were not initially determined to be concordant, an additional analysis using tissue BEAMing as an orthogonal tissue test was performed by Sysmex Inostics GmbH (Hamburg, Germany). The cutoff threshold for BEAMing analyses in tissue was set at 1% as a safeguard to avoid potential false-positive results caused by interpatient contamination resulting from tissue sectioning.

1. Tabernero J, Lenz H-J, Siena S. Analysis of circulating DNA and protein biomarkers to predict the clinical activity of regorafenib and assess prognosis in patients with metastatic colorectal cancer: a retrospective, exploratory analysis of the CORRECT trial. Lancet Oncol. 2015 Aug;16(8):937–48.
