# Supplementary material for: Plasma ctDNA RAS mutation analysis for the diagnosis and treatment monitoring of metastatic colorectal cancer patients
Source: Ann Oncol. 2017 Apr 13;28(6):1325–32. doi: 10.1093/annonc/mdx125 (PMC5834035; doi:10.1093/annonc/mdx125)
Supplement: mdx125_supp [file mdx125_supp.zip › Supplementary table 1_review.docx]

**Supplementary table S1: Baseline clinico-pathological characteristics**

| **Patient Characteristics** | **Total population [*N* (%)]** | | **Plasma *RAS* WT patients [*n* (%)]** | | **Plasma *RAS* MUT patients [*n* (%)]** | **MAF [median (IQR)]** | ***p*** |
| --- | --- | --- | --- | --- | --- | --- | --- |
| Patients (*n*) | | 115 | | 56 | 59 | 59 |  |
| Age (years) [median (IQR)] | | 67.8  (60.3-76.5) | | 68.2  (61.0-68.7) | 65.6  (59.4-76.8) | 65.6  (59.4-76.8) |  |
| **Age group** | |  | |  |  |  | 0.290 |
| <65 | | 45  (39.1%) | | 20  (35.7%) | 25  (42.4%) | 5.348  (0.316-13.185) |  |
| >65 | | 70  (60.9%) | | 36  (64.3%) | 34  (57.6%) | 1.321  (0.253-5.412) |  |
| **Gender** | |  | |  |  |  | 0.357 |
| Male | | 81  (70.4%) | | 38  (67.9%) | 43  (72.9%) | 1.270  (0.284-9.891) |  |
| Female | | 34  (29.6%) | | 18  (32.1%) | 16  (27.1%) | 3.403  (0.306-12.564) |  |
| **Stage at diagnoses** | |  | |  |  |  | 0.196 |
| II / III | | 21  (18.3%) | | 11  (19.6%) | 10  (16.9%) | 0.502  (0.117-4.806) |  |
| IV | | 94  (81.7%) | | 45  (80.4%) | 49  (83.1%) | 2.317  (0.316-11.942) |  |
| **Primary site of disease** | |  | |  |  |  | 0.590 |
| Right colon | | 33  (28.7%) | | 14  (25.0%) | 19  (32.2%) | 1.420  (0.149-10.740) |  |
| Left colon /Rectum | | 80  (69.6%) | | 41  (73.2%) | 39  (66.1%) | 2.317  (0.393-11.295) |  |
| Unknown | | 2  (1.7%) | | 1  (1.8%) | 1  (1.7%) |  |  |
| **Primary tumor resected** | |  | |  |  |  | 0.584 |
| No | | 52  (45.2%) | | 25  (44.6%) | 27  (45.8%) | 4.026  (0.358-12.037) |  |
| Yes | | 63  (54.8%) | | 31  (55.4%) | 32  (54.2%) | 1.558  (0.284-8.403) |  |
| **Systemic treatment 1 month before ctDNA** | |  | |  |  |  | **0.007** |
| No | | 92  (80.0%) | | 43  (76.8%) | 49  (83.0%) | 4.178  (0.451-12.620) |  |
| Yes | | 19  (16.5%) | | 11  (19.6%) | 8  (13.6%) | 0.173  (0.074-1.156) |  |
| Unknown | | 4  (3.5%) | | 2  (3.6%) | 2  (3.4%) |  |  |
| **Previous lines of treatment** | |  | |  |  |  | 0.585 |
| 0 | | 95  (82.6%) | | 43  (76.8%) | 52  (88.1%) | 1.63  (0.27-11.02) |  |
| 1 | | 16  (13.8%) | | 11  (19.6%) | 5  (8.5%) | 0.56  (0.06-18.2) |  |
| 2 | | 4  (3.5%) | | 2  (3.6%) | 2  (3.4%) | 11.22 |  |
| **Tumor site biopsy** | |  | |  |  |  | 0.885 |
| Primary | | 84  (73.0%) | | 40  (71.4%) | 44  (74.6%) | 1.345  (0.149-11.295) |  |
| Metastasis | | 12  (10.4%) | | 7  (12.5%) | 5  (8.5%) | 2.780  (0.840-4.026) |  |
| Unknown | | 19  (16.5%) | | 9  (16.1%) | 10  (16.9%) |  |  |
| **Number of metastatic sites** | |  | |  |  |  | 0.790 |
| 1 | | 66  (57.4%) | | 29  (51.8%) | 37  (62.7%) | 1.270  (0.246-11.42) |  |
| 2 | | 38  (33.0%) | | 20  (35.7%) | 18  (30.5%) | 3.403  (0.451-11.139) |  |
| 3 or more | | 11  (9.6%) | | 7  (12.5%) | 4  (6.8%) | 0.712  (0.201-11.220) |  |
| **Metastasis Location** | |  | |  |  |  |  |
| Liver | | 86  (74.8%) | | 41  (73.2%) | 45  (76.3%) | 4.806  (0.565-13.185) | **0.001** |
| No liver met | | 29  (25.2%) | | 15  ()26.8% | 14  (23.7%) | 0.203  (0.097-1.270) |  |
| Lung | | 44  (38.3%) | | 22  (39.3%) | 22  (37.3%) | 1.633  (0.253-11.139) | 0.718 |
| No Lung met | | 71  (61.7%) | | 34  (60.7%) | 37  (62.7%) | 2.052  (0.315-11.942) |  |
| Peritoneum | | 27  (23.5%) | | 15  (26.8%) | 12  (20.3%) | 0.383  (0.130-1.558) | 0.068 |
| No peritoneum met | | 88  (76.5%) | | 41  (73.2%) | 47  (79.7%) | 4.026  (0.512-12.283) |  |
| Node | | 17  (14.8%) | | 14  (25.0%) | 3  (5.1%) | 0.161  (0.124-11.696) | 0.220 |
| No node met | | 98  (85.2%) | | 42  (75.0%) | 56  (94.9%) | 1.949  (0.316-11.696) |  |
| Others | | 7  (6.1%) | | 3  (5.4%) | 4  (6.8%) | 0.201  (0.084-2.864) | 0.116 |
| No others met | | 108  (93.9%) | | 53  (94.6%) | 55  (93.2%) | 2.052  (0.383-11.696) |  |
| **Lung metastasis** | |  | |  |  |  | 0.129 |
| No Lung metastasis | | 71  (61.7%) | | 34  (60.7%) | 37  (62.7%) | 2.052  (0.315-11.942) |  |
| Only lung metastasis | | 9  (7.8%) | | 5  (8.9%) | 4  (6.8%) | 0.335  (0.074-0.614) |  |
| Lung and other metastasis | | 35  (30.4%) | | 17  (30.4%) | 18  (30.5%) | 3.403  (1.109-11.450) |  |
| **Peritoneum metastasis** | |  | |  |  |  | 0.056 |
| No Peritoneum metastasis | | 88  (76.5%) | | 41  (73.2%) | 47  (79.7%) | 4.026  (0.512-12.283) |  |
| Only peritoneum metastasis | | 11  (9.6%) | | 6  (10.7%) | 5  (8.5%) | 0.100  (0.097-0.246) |  |
| Peritoneum and other metastasis | | 16  (13.9%) | | 9  (16.1%) | 7  (11.9%) | 1.109  (0.383-6.253) |  |
| **Concordance** | |  | |  |  |  |  |
| Yes | | 107  (93.0%) | | 54  (96.4%) | 53  (89.8%) | 2.317  (0.451-11.450) | 0.193 |
| No | | 8  (7.0%) | | 2  (3.6%) | 6  (10.2%) | 0.281  (0.128-0.840) |  |
