# Supplementary material for: Plasma ctDNA RAS mutation analysis for the diagnosis and treatment monitoring of metastatic colorectal cancer patients
Source: Ann Oncol. 2017 Apr 13;28(6):1325–32. doi: 10.1093/annonc/mdx125 (PMC5834035; doi:10.1093/annonc/mdx125)
Supplement: mdx125_supp [file mdx125_supp.zip › Supplementary Table S2.docx]

**Supplementary Table S2. Emergence of *RAS* mutations at the time of progression to anti-EGFR treatment in 18 patients with *RAS* wild-type tumors at baseline.** All patients achieved partial response or disease stabilization for at least 16 weeks under EGFR blockade, administered alone or in combination with standard chemotherapy indicate

| **Patient nº** | **RAS mutation (MAF, %)** | | | | | |
| --- | --- | --- | --- | --- | --- | --- |
|  | KRAS exon2 | KRAS exon3 | KRAS exon4 | NRAS exon2 | NRAS exon3 | NRAS exon4 |
| **16** |  |  |  |  |  |  |
| **17** | K12 (0.172) |  |  |  |  |  |
| **18** |  |  |  |  | N61 (0.024) |  |
| **19** |  |  |  |  |  |  |
| **20** | K12 (0.21) | K61 (0.038) |  | N12 (0.046) | N61 (0.037) |  |
| **21** |  |  |  |  |  |  |
| **22** | K12 (5.51)  K13 (0.153) |  |  | N13 (0.06) | N61 (0.037) |  |
| **23** |  |  |  |  |  |  |
| **24** |  |  |  |  |  |  |
| **25** | K12 (0.137) | K61 (1.061) |  |  |  |  |
| **26** | K12 (0.168) |  |  |  |  |  |
| **27** |  |  | K146 (24.957) |  |  |  |
| **28** |  |  |  |  |  |  |
| **29** |  |  |  |  |  |  |
| **30** |  |  |  |  |  |  |
| **31** |  |  |  |  |  |  |
| **33** |  |  |  |  |  |  |
| **34** |  |  |  |  |  |  |

MAF, mutant allele fraction
