# Supplementary material for: Plasma ctDNA RAS mutation analysis for the diagnosis and treatment monitoring of metastatic colorectal cancer patients
Source: Ann Oncol. 2017 Apr 13;28(6):1325–32. doi: 10.1093/annonc/mdx125 (PMC5834035; doi:10.1093/annonc/mdx125)
Supplement: mdx125_supp [file mdx125_supp.zip › Supplementary table S3_review.docx]

**Supplementary table S3**. **Longitudinal analysis of circulating *RAS* mutations in 21 patients during treatment with chemotherapy +/- antiangiogenic or anti-EGFR**. Frequency of circulating *RAS* mutant alleles is shown at specific time points and percentage MAF variation between baseline and first CT scan performed at 8-12 weeks of treatment. Treatment received and best response is described for each patient.

| **Pt nº (baseline mut)** | **MAF (%)** | | | | | **Treatment** | **Best Response** |
| --- | --- | --- | --- | --- | --- | --- | --- |
|  | **Baseline** | **Week 4** | **Week 8-12:**  **first CT scan** | **CT Scan: PD** | **% Variation at first CT scan** |  |  |
| 1 (N61) | 6.1463 | 0.044 | 0.072 | 3.871 | - 98.83% | FOLFOX_Bevacizumab | SD |
| 2 (K146) | 0.3153 | 0.54 | 1.35 | - | 328% | Cetuximab | PD |
| 3 (N61) | 5.17 | ND | 0.41 | 10.74 | - 92.07% | Capecitabine_Bevacizumab | SD |
| 4 (K13) | 4.03 | 17.18 | 19.18 | - |  | FOLFOX | PD |
| 5 (N61) | 2.052 | 0 | 0 | - | - 100% | FOLFOX | PD |
| 6 (K146) | 12.62 | 8.9% | EXITUS | - |  | Capecitabine | PD |
| 7 (K13) | 0.2458 | 2.9 | 0.429 | - | 74.53% | FOLFOX-Panitumumab | PD |
| 8 (K146) | 6.8 | 0.949 | 0.107 | 4.46 | - 98.43% | FOLFOX_Bevacizumab | SD |
| 9 (N61) | 1.27 | 0 | 0 | 0.46 | - 100.00% | FOLFOX6 | SD |
| 10 (K12) | 0.161 | ND | 0 | ND | - 100.00% | FOLFOX_Bevacizumab | SD |
| 11 (K12) | 10.659 | ND | 0.038 | 2.554 | - 99.64% | FOLFOX_Bevacizumab | SD |
| 12 (K12) | 5.412 | 0 | 0 | ND | - 100.00% | XELOX | SD |
| 13 (K13) | 23.44 | ND | 0 | 9.56 | - 100.00% | Irinotecan | SD |
| 14 (K13) | 1.109 | ND | 0.913 | - | - 17% | FOLFIRI | PD |
| 15 (K12) | 1.8462 | 0.12 | 0 | 1.659 | - 100.00% | FOLFOX | PR |
| 16 (K12) | 0.4705 | 0 | ND | 14.475 |  | FOLFOX | PR |
| 17 (K13) | 4.8057 | 0.073 | 0 | 3.305 | - 100.00% | FOLFOX | PR |
| 18 (N61) | 23.2071 | 5.813 | ND | 14.817 |  | FOLFOX_Aflibercept | PR |
| 19 (K12) | 9.1229 | 3.71 | 11.195 | EXITUS | 22.71% | FOLFOX | SD |
| 20 (K12) | 0.675 | 0 | 0 | 0 | - 100.00% | Capecitabine | PR |
| 21 (K12) | 16.6623 | 0.879 | ND | 4.88 |  | FOLFOX_Aflibercept | SD |

MAF. mutant allele fraction; PD. progressive disease; PR. partial response. SD. stable disease
