# Supplementary material for: Plasma ctDNA RAS mutation analysis for the diagnosis and treatment monitoring of metastatic colorectal cancer patients
Source: Ann Oncol. 2017 Apr 13;28(6):1325–32. doi: 10.1093/annonc/mdx125 (PMC5834035; doi:10.1093/annonc/mdx125)
Supplement: mdx125_supp [file mdx125_supp.zip › Supplementary table S4.docx]

**Supplementary table S4**: *RAS* Mutations analyzed by OncoBEAM™ RAS CRC assay

| **KRAS** | | **NRAS** | |
| --- | --- | --- | --- |
| Exon | Mutation | Exon | Mutation |
| 2 | G12S | 2 | G12S |
|  | G12R |  | G12R |
|  | G12C |  | G12C |
|  | G12D |  | G12D |
|  | G12A |  | G12A |
|  | G12V |  | G12V |
|  | G13D |  | G13R |
| 3 | A59T |  | G13D |
|  | Q61L |  | G13V |
|  | Q61R | 3 | A59T |
|  | Q61H |  | Q61K |
| 4 | Q61H |  | Q61R |
|  | K117N |  | Q61L |
|  | K117N |  | Q61H |
|  |  |  | Q61H |
|  |  | 4 | K117N |
|  |  |  | K117N |
|  |  |  | A146T |
