# Supplementary figures and images for: Plasma ctDNA RAS mutation analysis for the diagnosis and treatment monitoring of metastatic colorectal cancer patients
Source: Ann Oncol. 2017 Apr 13;28(6):1325–32. doi: 10.1093/annonc/mdx125 (PMC5834035; doi:10.1093/annonc/mdx125)

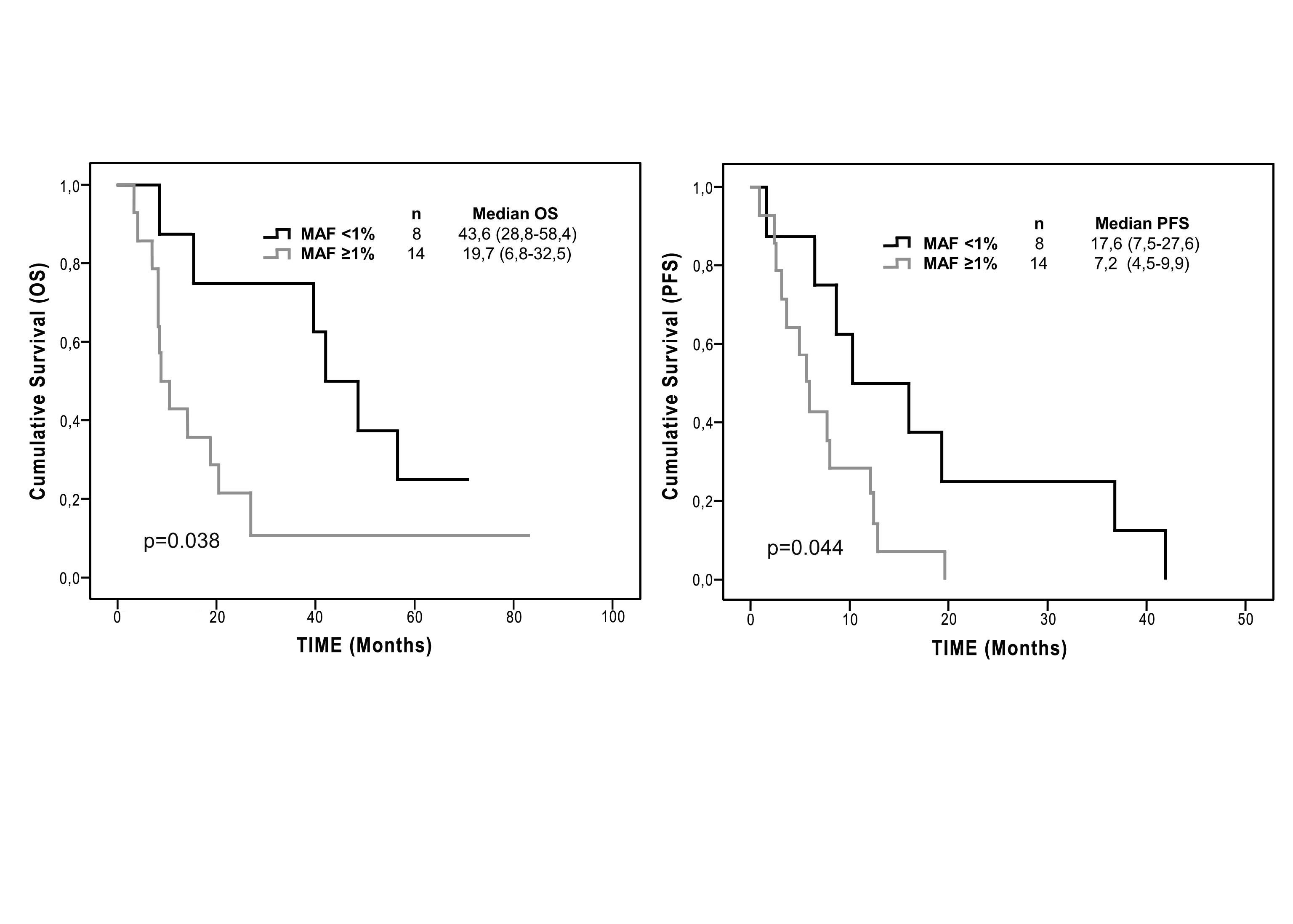

Supplement: mdx125_supp [file mdx125_supp.zip › Supplementary_figure2.tif]

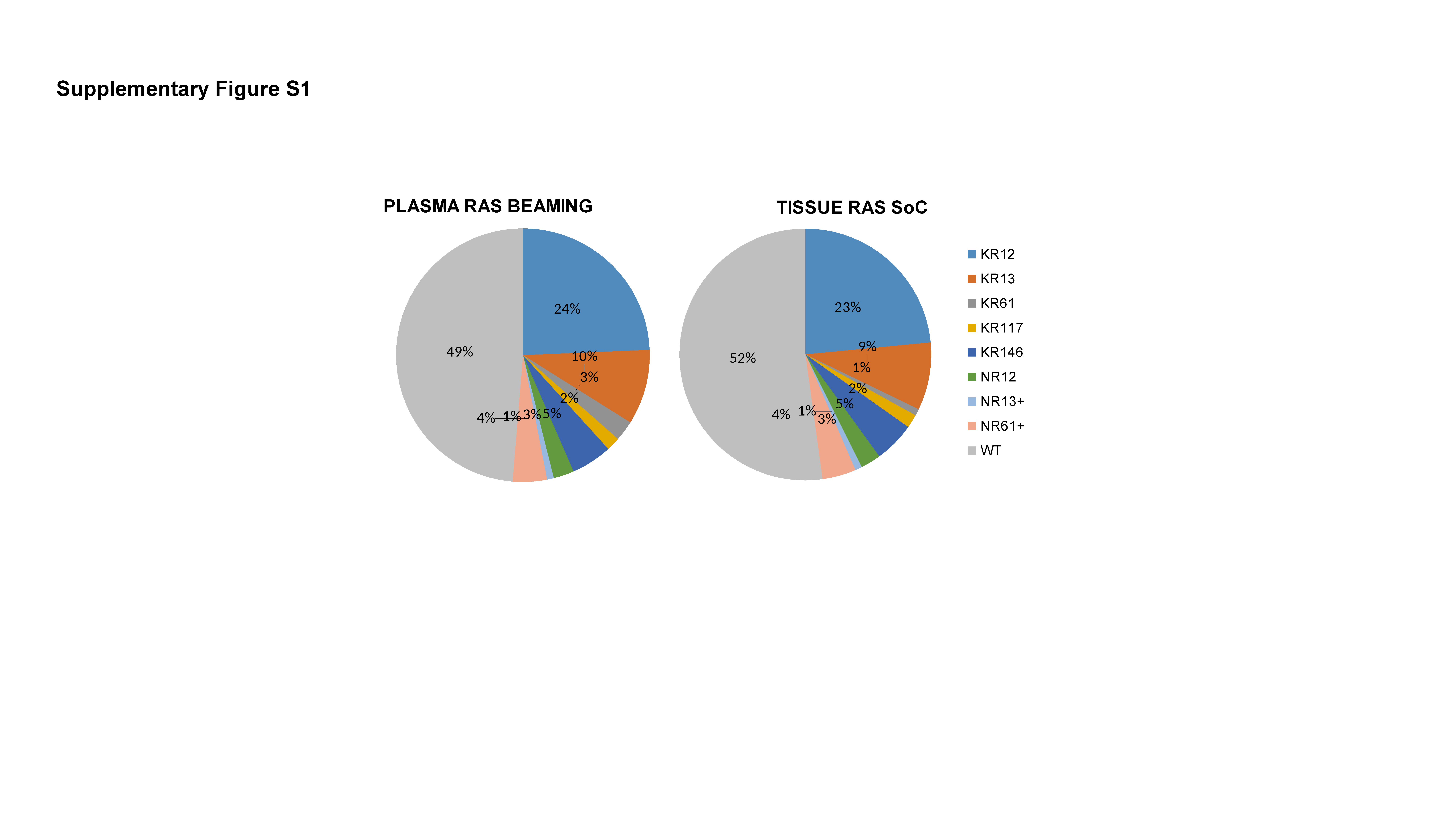

Supplement: mdx125_supp [file mdx125_supp.zip › Supplementary_figure1.tiff]
